# The Human Endogenous Protection System against Cell-Free Hemoglobin and Heme Is Overwhelmed in Preeclampsia and Provides Potential Biomarkers and Clinical Indicators

**DOI:** 10.1371/journal.pone.0138111

**Published:** 2015-09-14

**Authors:** Magnus Gram, Ulrik Dolberg Anderson, Maria E. Johansson, Anneli Edström-Hägerwall, Irene Larsson, Maya Jälmby, Stefan R. Hansson, Bo Åkerström

**Affiliations:** 1 Lund University, Department of Clinical Sciences Lund, Infection Medicine, Lund, Sweden; 2 Lund University, Department of Clinical Sciences Lund, Obstetrics and Gynecology, Lund, Sweden; Medical Faculty, Otto-von-Guericke University Magdeburg, Medical Faculty, GERMANY

## Abstract

Preeclampsia (PE) complicates 3–8% of all pregnancies and manifests clinically as hypertension and proteinuria in the second half of gestation. The pathogenesis of PE is not fully understood but recent studies have described the involvement of cell-free fetal hemoglobin (HbF). Hypothesizing that PE is associated with prolonged hemolysis we have studied the response of the cell-free Hb- and heme defense network. Thus, we have investigated the levels of cell-free HbF (both free, denoted HbF, and in complex with Hp, denoted Hp-HbF) as well as the major human endogenous Hb- and heme-scavenging systems: haptoglobin (Hp), hemopexin (Hpx), α_1_-microglobulin (A1M) and CD163 in plasma of PE women (n = 98) and women with normal pregnancies (n = 47) at term. A significant increase of the mean plasma HbF concentration was observed in women with PE. Plasma levels of Hp and Hpx were statistically significantly reduced, whereas the level of the extravascular heme- and radical scavenger A1M was significantly increased in plasma of women with PE. The Hpx levels significantly correlated with maternal blood pressure. Furthermore, HbF and the related scavenger proteins displayed a potential to be used as clinical biomarkers for more precise diagnosis of PE and are candidates as predictors of identifying pregnancies with increased risk of obstetrical complications. The results support that PE pathophysiology is associated with increased HbF-concentrations and an activation of the physiological Hb-heme defense systems.

## Introduction

Preeclampsia (PE) complicates 3–8% of all pregnancies and manifests clinically in the second half of gestation [1]. The classical findings that define PE are hypertension and proteinuria appearing after 20 weeks of gestation. PE is a potentially serious condition that in worst case can lead to eclampsia, characterized by general seizures and coma [2–4]. A related disease, the HELLP syndrome, (hemolysis, elevated liver enzymes and low platelets count) develops more rapidly and is accompanied with maternal hemolysis. Uniform classification of the different forms of hypertensive conditions during pregnancy is important in order to optimize patient management. To date several biomarkers have been suggested for screening in the first and second trimester, however none are yet recommended for screening in clinical practice [5]. Several biomarkers have also been suggested to support clinicians in their diagnostics and handling of the patients at term pregnancy [6–8].

The pathogenesis of PE is not fully understood but recent studies have described that extracellular fetal hemoglobin (HbF) is involved [9,10]. Using genomics and proteomics, Centlow et al showed an up-regulated gene expression of HbF and accumulation of cell-free HbF in the vascular lumen of term PE placentas [11]. May et al later showed, in the *ex vivo* human placenta perfusion system, that perfusion with cell-free hemoglobin (Hb) causes tissue damage and leakage of Hb over the placental barrier [12]. It was hypothesized that through the generation of reactive oxygen species (ROS), Hb induces oxidative damage to the placenta and a subsequent leakage over the blood-placental barrier [12]. In fact, Olsson et al [13] demonstrated that pregnant women diagnosed with PE have increased plasma levels of cell-free HbF and adult hemoglobin (HbA) at term and Anderson et al demonstrated that the serum levels of HbF were elevated already in the first trimester of pregnant women that later developed PE [14]. Furthermore, in term pregnancies the plasma concentration of cell-free total Hb (HbF + HbA) was shown to correlate with blood pressure, *i*.*e*. the severity of the disease [13].

Hemoglobin is a tetramer consisting of four globin subunits each carrying a heme-group in its active center [15]. In adults the most common Hb isoform is HbA that consists of two α- and two β-subunits (α_2_β_2_). In the fetus, the HbF isoform is the predominant type and consists of two α-chains and two γ-chains (α_2_γ_2_). Heme consists of an organic ring-structure, protoporphyrin IX, which chelates a ferrous (Fe^2+^) iron atom with high affinity for free oxygen (O_2_). Ferrous Hb binding to O_2_ is denoted oxyHb. Autoxidation of oxyHb is a spontaneous intramolecular redox reaction eventually leading to production of ferric (Fe^3+^) Hb (metHb), ferryl (Fe^4+^) Hb, free heme and various ROS [16,17]. These compounds are chemically very reactive and have the potential to induce tissue damage and cell destruction by one-electron redox reactions with biomolecules. As described above, it was hypothesized that the increased concentrations of HbF in PE causes oxidative damage to the placenta and a subsequent leakage over the feto-maternal barrier into the maternal circulation [12–14]. As a consequence, the vascular endothelium is damaged and eventually glomerular endotheliosis are developed, a pathognomonic kidney damage that occurs in PE. This damage eventually contributes to development of hypertension and proteinuria, the clinical hallmarks of PE.

Hb is normally found enclosed by the erythrocyte membranes. The autoxidation of intracellular oxyHb and downstream free radical formation is prevented mainly by superoxide dismutase (SOD), catalase and glutathione peroxidase (GPx) [18,19]. However, significant amounts of Hb escape from the erythrocytes under healthy conditions and massive amounts can be released during pathological conditions involving hemolysis, causing severe organ damage. Therefore a number of defense mechanisms have evolved both in plasma and in the extravascular compartments to counteract the damage caused by cell-free Hb.

Haptoglobin (Hp) is perhaps the most well investigated Hb-clearing molecule. It binds cell-free Hb in plasma [20,21] and the resulting Hp-Hb complex is cleared from blood via binding to the macrophage receptor CD163 [22]. The Hp molecule consists of two chains, α and β, and two allelic variants of the α-chains exist, α1 and α2. As a result, three phenotypic variants occur in the human population, Hp 1–1, Hp 2–2, and the allelic mixture, called Hp 1–2. Free heme in blood is sequestered by hemopexin (Hpx) [23,24] and the Hpx-heme complex is cleared from the circulation by the hepatocyte receptor CD91 [25]. In the extravascular compartment, cellular heme oxygenase (HO) is the most essential heme degrading protein, converting heme to free iron, biliverdin and CO [26,27]. Furthermore, the plasma- and extravascular reductase and heme- and radical scavenger α_1_-microglobulin (A1M) binds and degrades free heme and can reduce metHb [28–30]. A1M also acts as an antioxidant by reducing and covalently binding the downstream ROS and radicals generated by cell-free Hb [31–34]

In this study we have employed PE as a model disease to study the response of the cell-free Hb-defense network in a pathological situation with prolonged hemolysis. Thus, we have investigated the levels of cell-free HbF (both free, denoted HbF, and in complex with Hp, denoted Hp-HbF) as well as the concentrations of the major human endogenous Hb-scavenging systems: Hp, Hpx, A1M and CD163. The results confirm that PE is associated with increased HbF-concentrations and an activation of the physiological Hb-heme defense systems. The results also suggest that the components of the Hb-Hp-Hpx-A1M network may be employed as diagnostic biomarkers and are potential clinical tools for PE and perinatal pregnancy outcome, individually or in various combinations.

## Materials and Methods

### Patients and demographics

In an on-going prospective Swedish cohort study, women diagnosed with PE, collected 2003–2011, and matched normal pregnancies (controls), collected during the same period, were retrospectively selected from our biobank. In total, 150 pregnant women were included in the study. Exclusion criteria were gestational hypertension, essential hypertension and gestational diabetes. In total 5 cases were excluded and are therefore not included in any of the Tables and Figures. Out of the 145 remaining patients, 98 had PE (cases) and 47 were normal pregnancies (controls). Patient demographics are described in Tables 1 and 2.

**Table 1:** Description of pregnancies.

Outcome	Normal pregnancy (Control; n = 47)	Preeclampsia (n = 98)	Early onset PE^1^ (n = 22)	Late onset PE^2^ (n = 74)
Age	29 (28–30)	31** (30–32)	32 **NS** (30–34)	30 **NS** (29–32)
BMI (kg/m^2^)	25.0 (23.7–26.3)	26.1 **NS** (25.1–27.0)	27.1 **NS** (24.3–29.9)	25.9 **NS** (24.9–26.9)
Parity (n)	0.2 (0.02–0.32)	0.5* (0.28–0.64)	0.82* (0.23–1.41)	0.37* (0.20–0.54)
Systolic BP^3^ (mmHg)	123 (120–126)	161** (157–165)	176** (167–185)	157** (153–160)
Diastolic BP^4^ (mmHg)	77 (75–79)	101** (99–103)	108** (103–112)	99** (97–101)
Proteinuria (g/L)	0.02 (0.00–0.04)	2.32** (2.02–2.61)	3.35** (2.68–4.02)	2.08** (1.77–2.39)
Gestational age at delivery (days)	282 (279–285)	256** (250–262)	212** (199–225)	269** (265–273)
Twin pregnancies (n)	0	8 (8%)	2 (9%)	6 (8%)
Gestational age at sampling (days)	281 (278–284)	253** (247–260)	208** (196–220)	266** (262–270)
IVF (n)	1 (2%)	8 (8%)	1 (5%)	7 (10%)
ICSI (= n)	1 (2%)	1 (1%)	1 (5%)	0
Egg donor recipient (n)	0	1 (1%)	0	1 (1%)
Medication to stimulate ovulation^5^ (n)	0	2 (2%)	0	2 (3%)

Patient demographics of PE cases and normal pregnancies (controls). Time of PE diagnosis was not known for 2 PE cases and therefore not included in the sub-classification of early and late onset PE. Values are shown as mean (95% confidence interval) or number (%). Statistical comparison vs. controls. p-value <0.05 is considered significant. **NS:** Not significant;

*:p = <0.05;

**:p = <0.001.

^1^ Early onset PE was defined as diagnosis before 34+0 weeks of gestation.

^2^ Late onset PE was defined as diagnosis before gestational week > 34+0.

^3^ Highest systolic blood pressure recorded within two weeks prior to delivery.

^4^ Highest diastolic blood pressure recorded within two weeks prior to delivery.

^5^ In one case not known, the other patient medicated with Pergotime.

**Table 2:** Outcome of pregnancies.

Outcome	Normal pregnancy (Control; n = 47)	Preeclampsia ( = 98)	Early onset PE^1^ (n = 22)	Late onset PE^2^ (n = 74)
Birth weight (gram)	3602 (3477–3726)	2834** (2621–3047)	1434** (1105–1764)	3213** (3045–3381)
Fetal gender (M:F)	23:24	46:49 **NS**	7:15 **NS**	37:34 **NS**
HELLP^3^	0	7 (7%)	3 (14%)	4 (5%)
Eclampsia^4^	0	5 (5%)	2 (9%)	3 (4%)
Induction (n)	10 (21%)	58** (59%)	2** (9%)	55** (75%)
Vaginal delivery (n)	35 (75%)	46* (47%)	3* (14%)	43* (59%)
Vacuum extraction (n)	8 (17%)	8* (8%)	0**	8** (11%)
Cesarean section (n)	12 (26%)	47** (48%)	18** (82%)	27** (37%)
SGA^5^	0	1 (1%)^5a^	0	1 (1%)
IUGR^6^	0	8 (8%)	5 (23%)	3 (4%)
Admitted to NICU^7^ (n)	2 (4%)	32** (36%)	14** (82%)	18*** (25%)
Neonatal death	0	1 (1%)	1 (5%)	0
Preterm^8^ (= n)	0	34** (35%)	20** (95%)	12** (16%)
APGAR10^9^	9.80 (9.64–9.96)	9.75 **NS** (9.62–9.89)	9.30* (8.80–9.70)	9.90 **NS** (9.70–10.0)

Patient demographics of PE cases and normal pregnancies (controls). Values are shown as mean (95% confidence interval) or number (%). Statistical comparison vs. controls. p-value <0.05 is considered significant. **NS:** Not significant;

*:p = <0.05;

**:p = <0.001.

^1^ Early onset PE was defined as diagnosis before 34+0 weeks of gestation.

^2^ Late onset PE was defined as diagnosis before gestational week > 34+0.

^3^ HELLP syndrome (Hemolysis, Elevated Liver enzymes, Low Platelets) diagnosed according to Mississippi classification.

^4^ Eclampsia was defined as seizures occurring during pregnancy and after delivery in the presence of PE.

^5^ SGA (Small for Gestational Age) defined as growth curve on Ultrasonography constant below curve.

^5a^ Patient defined as both SGA and IUGR.

^6^ IUGR (Intra Uterine Growth Restriction) was defined as growth below -2 standard deviations (-22%) on Ultrasonography (equivalent to growth below 3^rd^ percentile).

^7^ NICU (Neonatal Intensive Care Unit).

^8^ Preterm was defined as delivery before 36+6 weeks of gestation (258 days).

^9^ APGAR (Appearance, Pulse, Grimace, Activity, Respiration) score at 10 minutes.

### Sample collection

Patient sampling was performed following written consent and the study was approved by the ethical committee review board for human studies in Malmö/Lund, Sweden. Maternal venous samples were taken prior to delivery (during the last 24 hours of pregnancy) at the Department of Obstetrics and Gynecology, Lund University Hospital, Sweden. Six-ml blood samples were collected into EDTA Vacuette® plasma tubes (Greiner Bio-One GmbH, Kremsmünster, Austria) and centrifuged at 2000 xg for 20 minutes at room temperature (RT). The plasma was then transferred into cryo tubes and stored in -80°C until time of analysis.

Preeclampsia was defined as *de novo* hypertension and proteinuria after 20 weeks of gestation with 2 readings at least 4 hours apart of blood pressure ≥140/90 mmHg and proteinuria ≥300 mg per 24 hours[35]. For quantification of proteinuria dipstick analysis was accepted if no other quantification was made. The PE group was further sub-classified as early-onset PE (diagnosis ≤ 34+0 weeks of gestation, n = 22) or late onset PE (diagnosis >34+0 weeks of gestation, n = 74). There were 2 cases of PE with unknown time of diagnosis, and therefore not included in the sub-analyses. The pregnancy outcome was retrospectively obtained from the patient charts.

### Reagents and proteins

HbF was purified from whole blood, freshly drawn from umbilical cord blood, as previously described [17]. Human γ-chains were prepared by dissociation of purified HbF with p-mercuribenzoate (Sigma-Aldrich, St-Louis, MO, USA) and acidic precipitation as described by Kajita et al [36] with modifications by Noble [37]. The absolute purity of HbF (from contamination with HbA) and of γ-chains (from contamination with α- and β-chains) was determined as previously described [13]. Human Hp-HbF was prepared by mixing human Hp (1–1; Sigma-Aldrich) with HbF in a 1:1 ratio, and purifying the complex from free Hp and HbF by FPLC size exclusion chromatography. Mouse monoclonal antibodies and rabbit polyclonal antibodies against HbF were prepared by AgriSera AB (Vännäs, Sweden) by immunization with human γ-chains. From the polyclonal antisera, HbF-specific antibodies were purified by immunoglobulin purification (by protein A-Sepharose chromatography, Sigma) followed by HbF-affinity chromatography and removal of unspecific HbA antibodies (by absorbing on an HbA-affinity chromatography). Rabbit anti-Hb IgG were purchased from DAKO (Glostrup, Denmark) and further purified by HbA-affinity chromatography. Antibodies (monoclonal and rabbit polyclonal IgG used as detection antibodies) were conjugated with horseradish peroxidase (Lightning-Link HRP, Innova Biosciences, Cambridge, UK) according to the manufacturer’s instruction. Human A1M was purified from urine as described by Åkerström et al [38]. Goat polyclonal antibodies against human A1M and goat anti-rabbit immunoglobulin were prepared as previously described [39].

### Fetal hemoglobin (HbF)-concentrations

A sandwich-ELISA was used for quantification of uncomplexed HbF in plasma. Ninety six-well microtiter plates were coated with anti-HbF antibodies (mouse monoclonal (no. 85); 4μg/ml in PBS) overnight at RT. In the second step, wells were blocked for 2 hours using blocking buffer (1% BSA in PBS), followed by an incubation with HbF calibrator or the patient samples for 2 hours at RT. In the third step, HRP-conjugated anti-HbF antibodies (mouse monoclonal (no. 417); diluted 1:5000), were added and incubated for 2 hours at RT. Finally, a ready-to-use 3,3′,5,5′-Tetramethylbenzidine (TMB, Life Technologies, Stockholm, Sweden) substrate solution was added. The reaction was stopped after 20 minutes using 1.0 M HCl and the absorbance was read at 450nm using a Wallac 1420 Multilabel Counter (Perkin Elmer Life Sciences, Waltham, MA, USA).

### Haptoglobin-fetal hemoglobin (Hp-HbF) concentrations

A sandwich-ELISA used for quantification of Hp-HbF was developed and displayed a high preference for Hp-HbF compared to uncomplexed HbF (>10x higher recovery of a Hp-HbF calibrator series compared to a HbF calibrator series with the same molar content of HbF). No cross-reactivity was observed with Hp or HbA. Ninety six-well microtiter plates were coated with anti-HbF antibodies (HbF-affinity purified rabbit polyclonal (“Bonita”); 4μg/ml in PBS) overnight at RT. In the second step, wells were blocked for 2 hours using blocking buffer (1% BSA in PBS), followed by an incubation with Hp-HbF calibrator or the patient samples for 2 hours at RT. In the third step, HRP-conjugated anti-Hb antibodies (HbA-affinity purified rabbit polyclonal; DAKO; diluted 1:5000), were added and incubated for 2 hours at RT. Finally, a ready-to-use TMB (Life Technologies) substrate solution was added. The reaction was stopped after 30 minutes using 1.0 M HCl and the absorbance was read at 450nm using a Wallac 1420 Multilabel Counter (Perkin Elmer Life Sciences).

### Total hemoglobin (Hb-Total)-concentrations

The concentration of total Hb in maternal plasma was determined using a Human Hb ELISA Quantification Kit from Genway Biotech Inc. (San Diego, CA, USA). The analysis was performed according to the manufacturer’s instructions and the absorbance was read at 450nm using a Wallac 1420 Multilabel Counter.

### α_1_-microglobulin (A1M)-concentrations

Radiolabelling of A1M with ^125^I (Perkin Elmer Life Sciences) was done using the chloramine T method. Protein-bound iodine was separated from free iodide by gel-chromatography on a Sephadex G-25 column (PD10, GE Healthcare, Stockholm, Sweden). A specific activity of around 0.1–0.2 MBq/μg protein was obtained. Radioimmunoassay (RIA) was performed by mixing goat antiserum against human A1M (“Halvan”; diluted 1:6000) with ^125^I-labelled A1M (appr. 0.05 pg/ml) and unknown patient samples or calibrator A1M-concentrations. After incubating overnight at RT, antibody-bound antigen was precipitated by adding bovine serum and 15% polyethylene glycol, centrifuged at 2500 rpm for 40 minutes, after which the ^125^I-activity of the pellets was measured in a Wallac Wizard 1470 gamma counter (Perkin Elmer Life Sciences).

### Haptoglobin (Hp)-concentrations

The concentration of Hp in maternal plasma was determined using a Human Hp ELISA Quantification Kit from Genway Biotech Inc. The analysis was performed according to the manufacturer’s instructions and the absorbance was read at 450nm using a Wallac 1420 Multilabel Counter.

### Hemopexin (Hpx)-concentrations

The concentration of Hpx in maternal plasma was determined using a Human Hpx ELISA Kit from Genway Biotech Inc. The analysis was performed according to manufacturer’s instructions and the absorbance was read at 450nm using a Wallac 1420 Multilabel Counter.

### Cluster of Differentiation 163 (CD163)-concentrations

The concentration of CD163 in maternal plasma was determined using a Human CD163 Duo Set from R&D Systems (Abingdon, UK). The analysis was performed according to the manufacturer’s instructions and the absorbance was read at 450nm using a Wallac 1420 Multilabel Counter.

### SDS-PAGE and Western blot

SDS-PAGE was performed using precast 4–20% Mini-Protean TGX gels from Bio-Rad (Hercules, CA, USA) and run under reducing conditions using molecular weight standard (precision protein plus dual marker) from Bio-Rad. The separated proteins were transferred to polyvinylidene difluoride (PVDF) or low fluorescence (LF) PVDF membranes (Bio-Rad). The membranes were then incubated with antibodies against Hp (rabbit polyclonal, 12μg/ml, DAKO). Western blot was performed using HRP-conjugated secondary antibodies (DAKO) and the chemiluminescent substrate Clarity Western ECL (Bio-Rad). The bands were detected in a ChemiDoc XRS unit (Bio-Rad).

### Statistical analysis

Statistical computer software Statistical Package for the Social Sciences (SPSS Inc., Chicago, IL) version 21 for Apple computers (Apple Inc., Cupertino, CA) and Origin 9.0 software (OriginLab Corporation, Northampton, MA, USA) were used to analyze the data.

ANOVA test was used to compare the groups for clinical parameters such as age, BMI, parity, systolic blood pressure, diastolic blood pressure, proteinuria, gestational age at delivery, birth weight, gestational age at time of sampling and APGAR score at10 minutes.

The Chi square test was used to compare the groups for fetal gender, labor induction, mode of delivery (e.g. vacuum extraction, caesarean section or vaginal delivery), need of neonatal intensive care unit (NICU) and preterm delivery.

Mean concentrations of the examined variables (henceforth referred to as biomarkers) were evaluated in women with PE compared to the control group using non-parametric statistics. A univariate logistic regression model was developed for the evaluated biomarkers. The gestational age at sampling was adjusted for in the logistic regression model. The biomarkers displaying a significant difference were further evaluated using Receiver Operational Curve (ROC-curve) by analyzing the area under the ROC-curve (AUC) as well as calculating the detection rates at different false positive levels. Parallel analysis was performed for each of the examined biomarker as well as different combinations of them. Furthermore, sub-group analysis of women with PE, *i*.*e*. early and late onset PE, compared to the control group was performed. The univariate logistic regression model was also used to further calculate how the biomarkers performed in terms of predicting fetal outcomes (*i*.*e*. admission to NICU and premature delivery and intrauterine growth restriction (IUGR)) and mode of delivery.

Correlation analysis (Pearson’s correlation coefficient) between biomarkers and diastolic- and systolic blood pressure was performed. A p-value of p≤0.05 was considered statistically significant in all tests.

## Results

### Patient characteristics

The characteristics of the included patients are shown in Tables 1 and 2. There was a significant difference in age, blood pressure, proteinuria, parity, gestational age at sampling, gestational age of delivery and birth weight between women diagnosed with PE and uncomplicated pregnancies (denoted controls). Furthermore, for parameters regarding maternal outcome (e.g. mode of delivery incl. induction and instrumental deliveries) as well as fetal outcome (e.g. admittance to NICU and prematurity) a statistically significant difference was observed between groups. A statistically significant difference in the 10 minutes APGAR score was observed between controls and early onset PE but not late onset PE. There was no statistical significant difference between the groups regarding BMI and fetal gender.

### Cell-free Hb

The concentration of cell-free HbF, Hp-HbF and Hb-Total were analyzed in all plasma samples from women with PE and controls (Table 3). A 4-fold increase of the HbF concentration was seen in the PE patients (p-value 0.01) as compared to the controls. When subdividing the PE group into early and late onset PE an almost 5-fold increase in the HbF concentration was observed in the early onset PE group as compared to controls (p-value 0.006). In the late onset PE group, an almost 4-fold increase was observed as compared to controls, but this was not statistically significant (p-value 0.17). A statistically significant increase in the mean Hp-HbF concentration was observed for women with PE as compared to controls (p-value 0.018). This difference was not found when comparing early and late onset PE separately with the control group, although a clear trend towards an increase could be seen in the early onset PE group (p-value 0.15).

**Table 3:** Biomarker results.

Biomarker	Normal pregnancy (Control; n = 47)	Preeclampsia (n = 98)	Early onset PE^1^ (n = 22)	Late onset PE^2^ (n = 74)
HbF (ng/ml)	3.85 (2.51–5.20)	15.26 (7.0–23.6) p = 0.01	18.72 (1.6–39.05) p = 0.006	14.60 (5.10–24.0) p = 0.17
Hp-HbF (μg/ml)	0.59 (0.003–1.18)	0.61 (0.31–0.90) p = 0.018	1.07 (-0.10–2.24) p = 0.15	0.48 (0.29–0.66) p = 0.02
Total-Hb (μg/ml)	277 (232–321)	285 (238–331) p = 0.53	290 (152–430) p = 0.80	284 (237–331) p = 0.73
Hp (mg/ml)	1.17 (1.04–1.30)	0.97 (0.75–1.19) p = <0.0001	1.34 (0.39–2.30) p = 0.067	0.89 (0.77–1.02) p = 0.001
CD 163 (μg/ml)	461 (408–512)	485 (445–527) p = 0.37	433 (324–543) p = 0.35	508 (465–551) p = 0.07
Hpx (mg/ml)	0.93 (0.88–0.98)	0.69 (0.66–0.73) p = <0.0001	0.69 (0.61–0.77) p<0.0001	0.69 (0.65–0.73) p<0.0001
A1M (μg/ml)	29.93 (27.89–31.97)	33.50 (31.90–35.10) p = 0.035	34.07 (30.31–37.83) p = 0.26	33.70 (31.90–35.50) p = 0.03

The mean concentrations of the biomarkers in the PE group and normal pregnancies (controls). Statistical comparison vs. controls. Significance was calculated with non-parametric statistics (Mann-Whitney). Values are mean values with (95% confidence interval). A p-value <0.05 was considered significant.

^1^ Early onset PE was defined as diagnosis before 34+0 weeks of gestation.

^2^ Late onset PE was defined as diagnosis before gestational week > 34+0.

No significant difference in Hb-Total concentration was observed between PE vs. controls (p-value 0.53) or between early (p-value 0.80) and late onset PE (p-value 0.73) vs. controls.

### Hp and CD163

Analysis of the Hp concentration in plasma showed a statistically significant decrease in Hp concentration in plasma samples of women with PE as compared to controls (p-value<0.0001). In addition, late onset PE displayed a significant decrease as compared to the controls (p-value 0.001). In contrast, early onset PE showed a slight but not statistically significant increase in Hp concentration as compared to the controls (p-value 0.067).

Soluble, shedded CD163, the macrophage receptor mediating elimination of the Hp-Hb complex, was analyzed in plasma [40–42]. The analysis showed a small but not statistically significant (p-value 0.37) increase in the PE group as compared to the controls (Table 3). Subdividing the PE group into early and late onset PE, a small, not statistically significant, increase was observed in the late onset PE group (p-value 0.07 vs. the controls) whereas a small, not statistically significant, decrease was observed in the early onset PE group (p-value 0.35 vs. the controls).

### Hpx

Analysis of the intravascular heme-scavenger protein Hpx showed a statistically significant decrease in plasma Hpx concentration of women with PE (p-value<0.0001) as compared to the controls (Table 3). Subdividing the PE group, displayed a statistically significant decrease in both the early (p-value<0.0001) and late onset PE (p-value<0.0001) PE groups as compared to the controls.

### A1M

Analysis of plasma levels of the heme- and radical scavenger A1M showed a statistically significant increase of plasma A1M concentration in women with PE (p-value 0.035) as compared to controls (Table 3). Subdividing the PE group, a statistically significant increase was observed in the late onset PE group (p-value 0.03) but not in the early onset PE group (p-value 0.26).

### Correlation cell-free HbF and Hp

**Fig 1:**
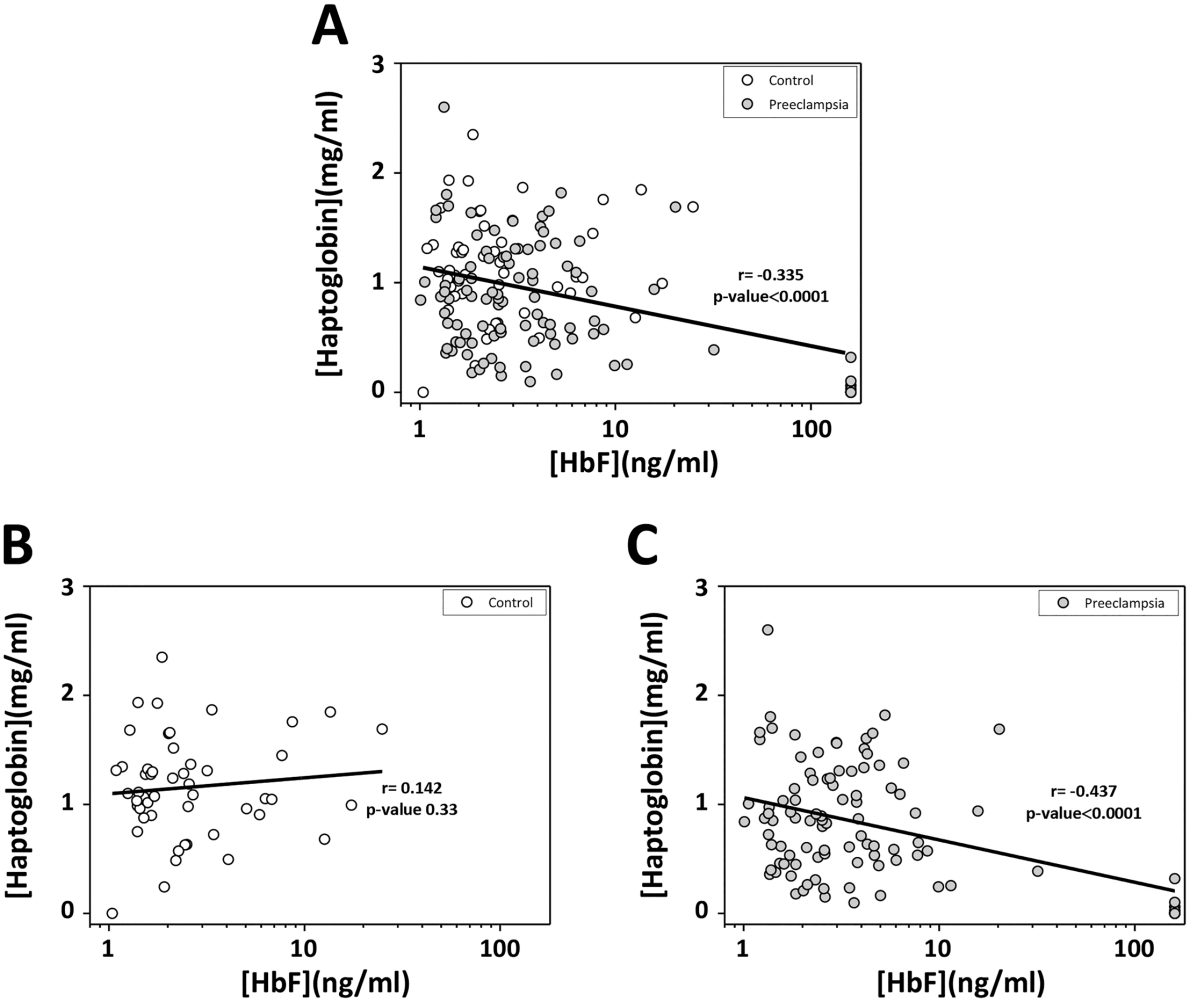
Correlation between cell-free HbF- and Hp concentrations. Samples were from normal pregnancies (Control) and women diagnosed with PE. The cell-free HbF plasma concentration of each patient sample (Control and PE) was plotted against the Hp plasma concentration (**A**). The cell-free HbF plasma concentration of Controls was plotted against the Hp plasma concentration (**B**). The cell-free HbF plasma concentration of women diagnosed with PE was plotted against the Hp plasma concentration (**C**). Associations between variables were assessed by linear regression analysis (Pearson’s).

The correlation between plasma cell-free HbF and Hp levels was evaluated. A negative correlation was found, *i*.*e*. an increased plasma cell-free HbF concentration was associated with a decreased plasma Hp concentration, when including all individuals, controls and women with PE (r = -0.335, p-value<0.0001, n = 145)(Fig 1A). Strikingly, when comparing the correlation in controls (Fig 1B) and women with PE (Fig 1C) separately, an increased negative correlation was observed for the PE group (r = -0.437, p-value<0.0001, n = 98) whilst in the control group a weak positive correlation was observed (r = 0.142, p-value 0.33, n = 47). Similar correlations were observed for Hp vs. Hp-HbF and Hp vs. Hb-Total, but none of them reached statistical significance (Hp vs. Hp-HbF r = -0.05, p-value 0.52; Hp vs. Hb-Total r = 0.03, p-value 0.73).

### Association between Hp isoform and cell-free HbF, Hpx and A1M

**Fig 2:**
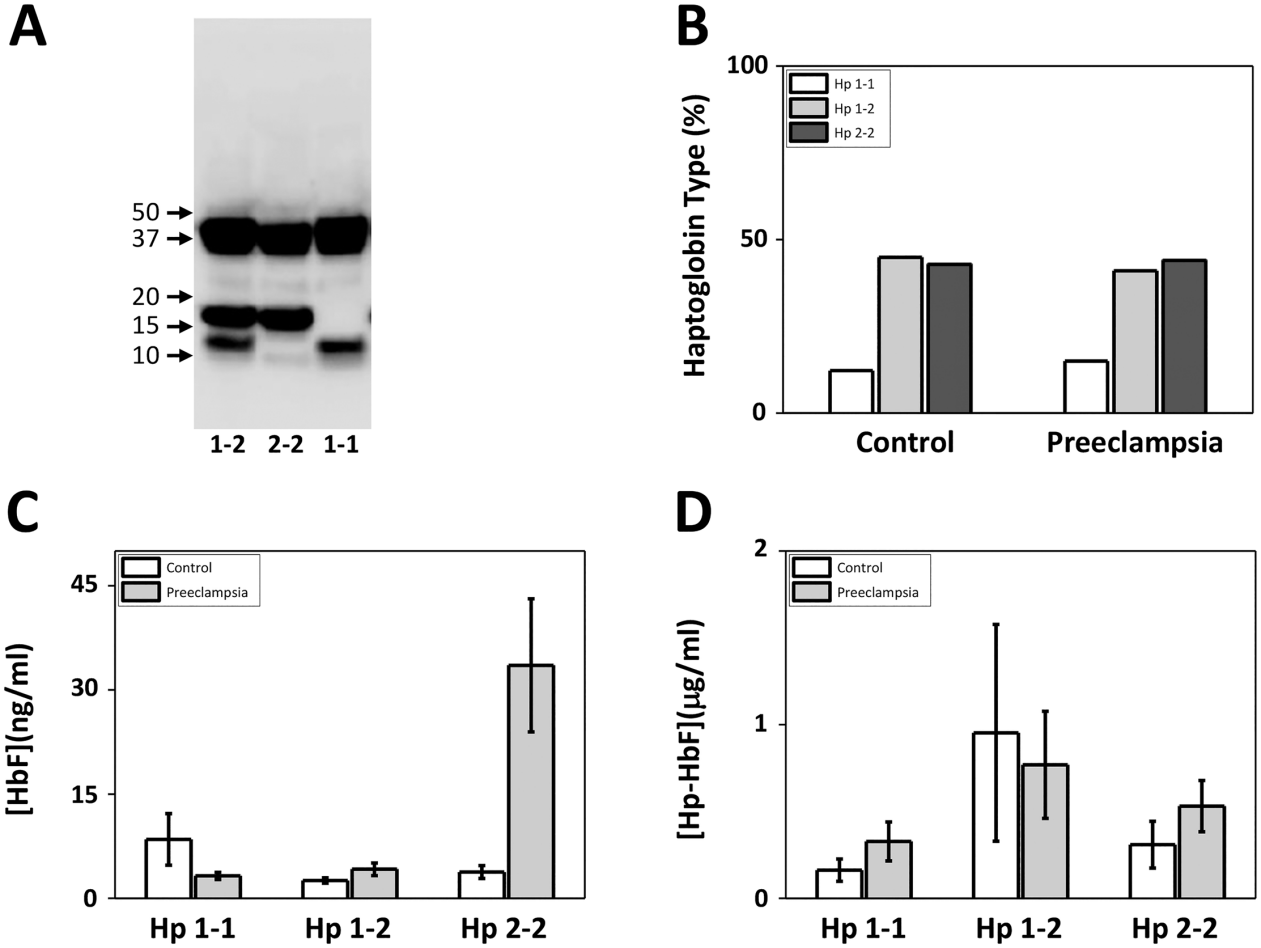
Correlation between Hp phenotype, cell-free HbF- and Hp-HbF concentration. Hp-phenotypes (1–1, 1–2 or 2–2) were investigated in plasma using SDS-PAGE and Western blot with anti-Hp antibodies as shown in the three patient examples (**A**) as described in Materials and Methods and the distribution of the different isoforms are presented as mean percentage of women with Hp 1–1, 1–2 and 2–2 for respective group (**B**). The plasma concentration of cell-free HbF (**C**) and Hp-HbF (**D**) are shown separately in patient samples with each Hp phenotype (Hp 1–1, 1–2 and 2–2). Results are presented as mean percentage of respective Hp phenotype (Hp 1–1, 1–2 and 2–2) in **B**. Results are presented as mean ± SEM plasma concentration of cell-free HbF and Hp-HbF in **C** and **D**.

We identified the predominant Hp-isoforms (1–1, 2–2, or both: 1–2) in the patient plasma samples using Western blot (Fig 2A). As seen in Fig 2B, a similar distribution of the different phenotypes were observed in both controls and PE, with a predominant presence of Hp 1–2 (C, 45%; PE, 41%) and 2–2 (C, 43%; PE, 44%) as compared to 1–1 (C, 12%; PE, 15%). Subdividing the PE group into early and late onset PE yielded a similar distribution 1–1 (early, 13%; late, 15%), 1–2 (early, 45%; late, 40%) and 2–2 (early, 42%; late, 45%). Furthermore, the association between the Hp-isoforms and the plasma levels of cell-free HbF and Hp-HbF were analyzed (Fig 2C and 2D). A striking increase in the concentration of cell-free HbF was observed in the Hp 2–2 group of women with PE (p-value 0.03)(Fig 2C). A smaller, but similar selective increase in the concentration of Hp-HbF was observed in the Hp 2–2 PE group as compared to controls (p-value 0.05)(Fig 2D). No significant association was observed between Hb-Total, Hp, CD163, Hpx and A1M to any of the Hp isoform.

### Correlation analysis between biomarkers and blood pressure

**Fig 3:**
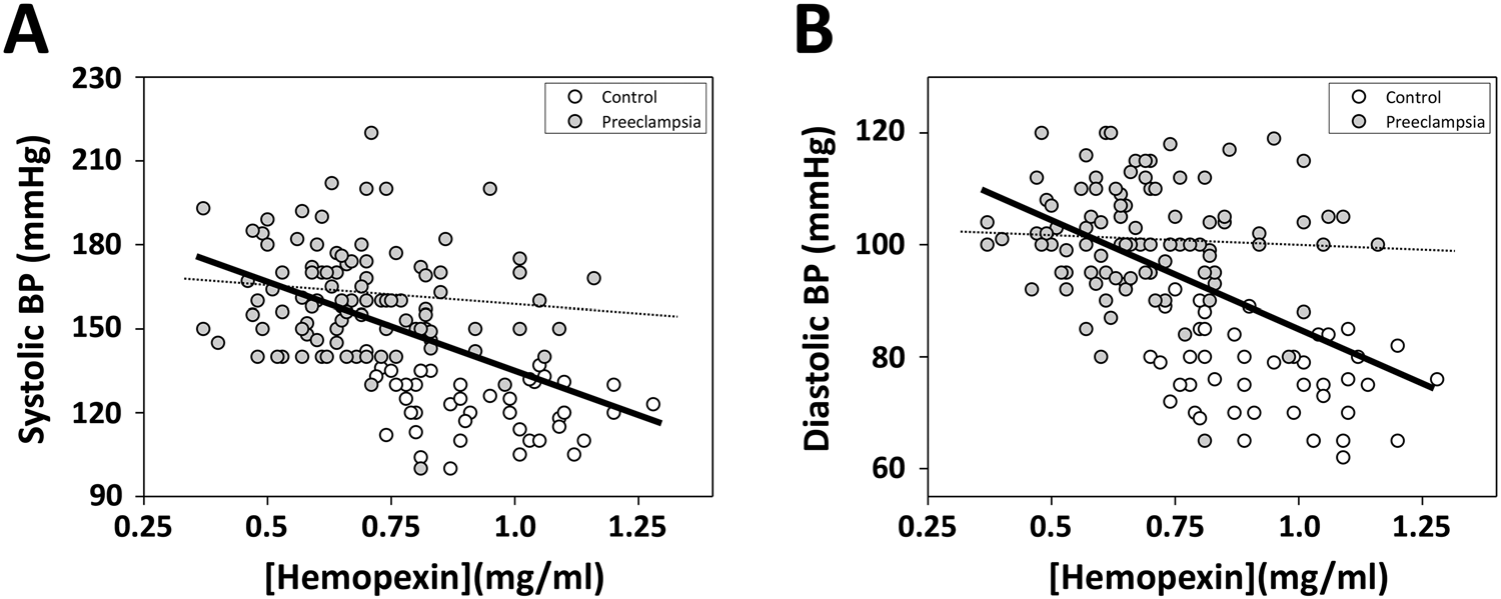
Correlation between Hpx concentration and systolic/diastolic blood pressure. Highest systolic (**A**) and diastolic (**B**) blood pressure (BP) measured within the last two weeks before delivery were plotted against the plasma concentration of Hpx. Correlation analysis of PE patients and controls using Pearson’s correlation coefficient between Hpx and blood pressure, systolic (**A**, **solid line**; r = -0.511, p-value<0.00001, n = 145) and diastolic (**B**, **solid line**; r = -0,520, p-value<0.00001, n = 145). Correlation analysis of PE patients only using Pearson’s correlation coefficient between Hpx and blood pressure, systolic (**A**, **dashed line**; r = -0,123, p-value 0.22, n = 98) and diastolic (**B**, **dashed line**; r = -0,058, p-value 0.57, n = 98).

Correlation analysis using Pearson’s correlation coefficient showed statistically significant inverse correlation between Hpx and blood pressure, both systolic (r = -0.511, p-value<0.00001, n = 145) and diastolic (r = -0,520, p-value<0.00001, n = 145)(Fig 3). Furthermore, correlation analysis of PE patients only displayed a slight but not statistically significant inverse correlation between Hpx and blood pressure (systolic, r = -0,123, p-value 0.22, n = 98; diastolic, r = -0,058, p-value 0.57, n = 98). No statistical significant correlation was observed for any of the other biomarkers in relation to the blood pressure.

### Evaluation of biomarkers as diagnostic markers of PE

**Fig 4:**
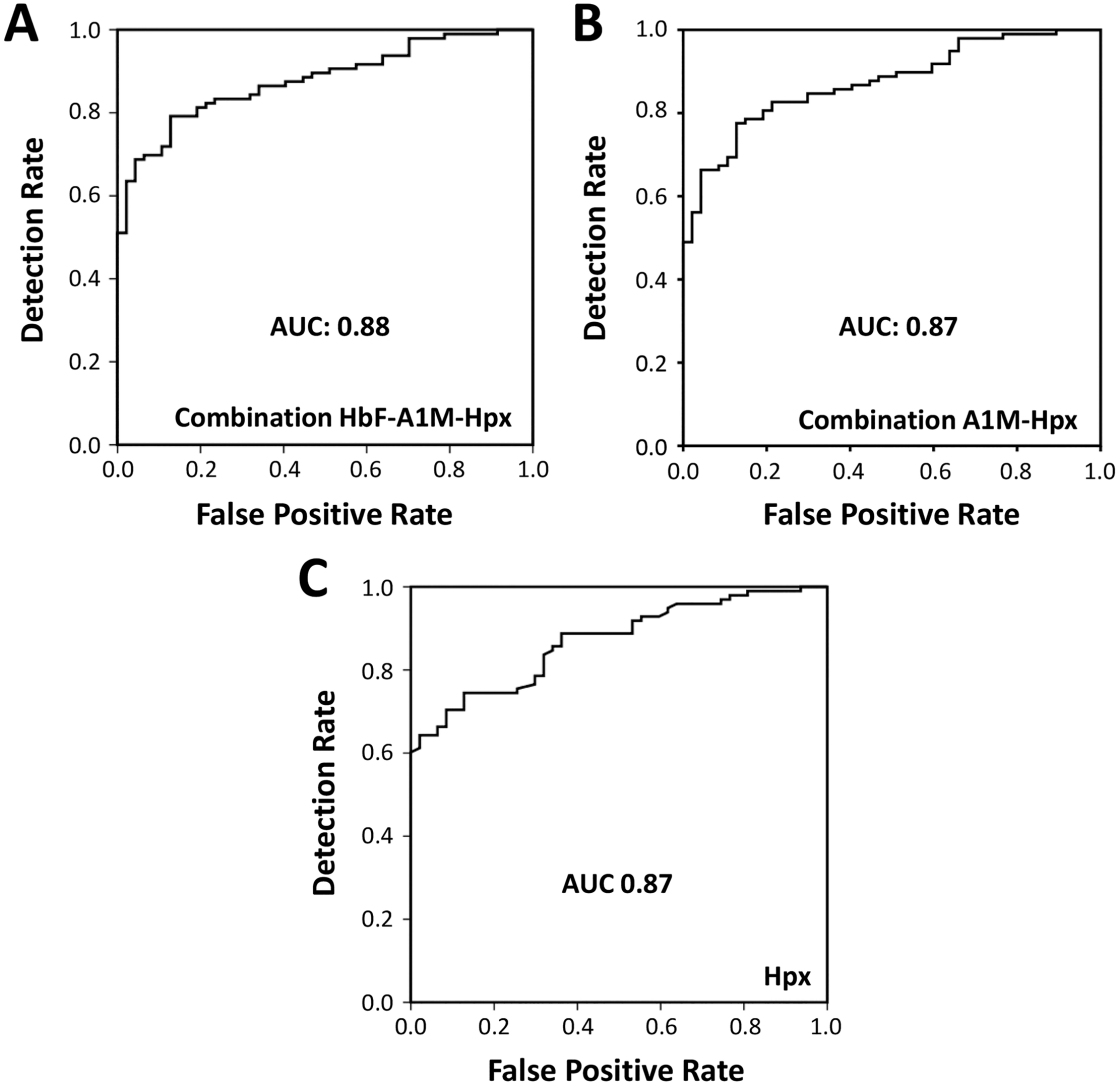
Receiver operating characteristic (ROC) curves. ROC curves showing sensitivity and specificity for the combination of HbF, A1M and Hpx (**A**), Hpx and A1M (**B**) and Hpx (**C**). Area under curve (AUC) is 0.88 for the combination of HbF, A1M and Hpx, 0.92 for the combination of A1M and Hpx and 0.87 for Hpx.

A logistic regression model was used to evaluate the usefulness of the described biomarkers as diagnostic markers of PE. By comparing women with PE vs. controls, a significant difference was detected for HbF (p-value 0.02), A1M (p-value 0.008) and Hpx (p-value<0.0001) but not for Hp (p-value 0.21) and CD163 (p-value 0.42). Each of the significantly altered biomarkers were able to diagnose PE (adjusted for gestational age) but Hpx showed the highest level of significance and a diagnostic detection rate of 64% at a false positive rate of 5% with an AUC of 0.87 (Table 4, Fig 4C). The combination of Hpx, A1M and HbF was not statistically significant (p-value for HbF 0.08) but displayed a diagnostic detection rate of 69% at a false positive rate of 5% with an AUC of 0.88 (Table 4, Fig 4A). The combination Hpx and A1M was statistically significant (p-value 0.05 for both biomarkers) and showed a diagnostic detection rate of 66% at a false positive rate of 5% and an AUC of 0.87 (Table 4, Fig 4B).

**Table 4:** Biomarker detection rates.

False positive rate	HbF combined with A1M and Hpx^1^	A1M combined with Hpx^2^	Hpx
5%	69%	66%	64%
10%	69%	67%	70%
20%	81%	81%	75%
30%	83%	85%	79%
AUC	0.88	0.87	0.87

Detection rates at fixed positive values for the combination of 1) HbF, A1M and Hpx, 2) A1M and Hpx and 3) Hpx alone. Detection rates for PE at different false positive rates and AUC for the ROC curve. Calculations are for all PE vs. controls.

^1^ Based on logistic regression including all three parameters.

^2^ Based on logistic regression including both parameters.

### Correlation with fetal and maternal outcomes

We further evaluated whether the biomarkers correlated with fetal and maternal outcomes (Table 5). A logistic regression model was used and the fetal outcome variables were: admission to NICU, presence of IUGR and premature birth. The maternal outcome variables were induction of labor, delivery by cesarean section and instrumental deliveries. The biomarkers HbF (p-value 0.001), Hpx (p-value 0.008) and Hp (p-value 0.03) each showed a association with “admission to NICU”. However, in a combined logistic regression model they were not statistically significant. The biomarkers Hpx (p-value 0.0003, AUC = 0.71) and CD163 (p-value 0.03, AUC = 0.61) showed a association with premature delivery. Furthermore, the combination of Hpx and CD163 also displayed a statistically significant correlation with premature delivery (p-value 0.001 and p-value 0.025, AUC 0.72).

Hpx displayed a statistically significant association with the risk of cesarean section (p-value 0.009, AUC 0.62). No further correlation was found between the evaluated biomarkers and maternal outcomes.

**Table 5:** Prediction of fetal and maternal outcomes.

Admittance to NICU	Significance	AUC
HbF	0.001	0.69
Hp	0.03	0.62
Hpx	0.008	0.66
**Prematurity**		
Hpx	0.001	0.70
CD 163	0.04	0.61
Combination Hpx + CD 163	0.001 0.025	0.72
**Cesarean section**		
Hpx	0.009	0.62

Area Under the ROC-curves (AUC) for fetal outcomes (admittance to Neonatal Intensive Care Unit (NICU) and prematurity) and maternal outcomes (risk of cesarean section). The fetal outcome IUGR and the maternal outcomes induction of labor and vacuum extraction were not significantly related to any of the biomarkers. All calculations were based on univariable logistic regression analysis.

## Discussion

**Fig 5:**
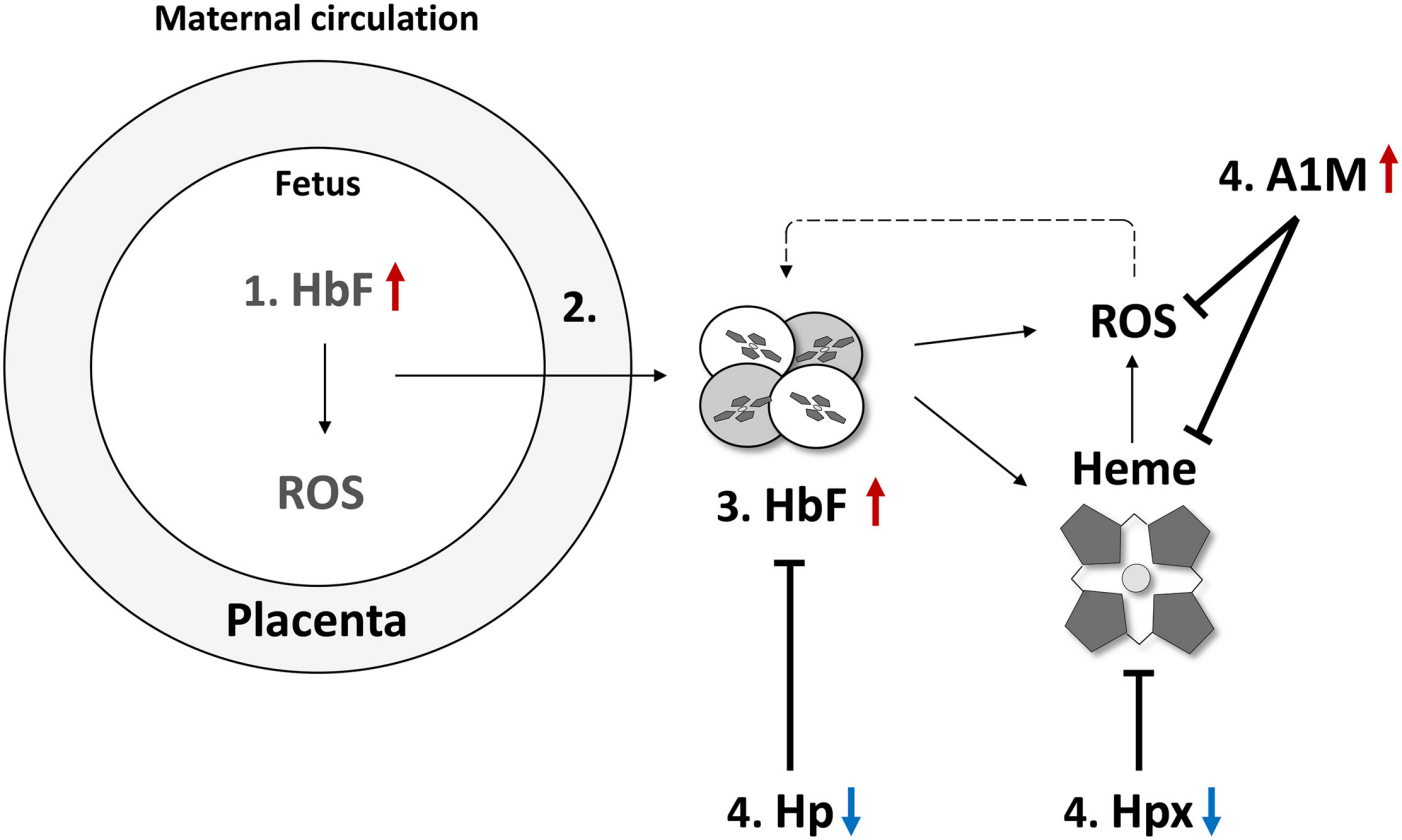
Schematic representation of the tentative chain of events involving HbF, Hp, Hpx, A1M and ROS and leading to PE. The figure shows a schematic placenta with impaired feto-maternal barrier function causing leakage of placenta factors. 1: Early events in the placenta induce an upregulation of the placenta HbF genes and protein and ROS. 2: Oxidative damage and leakage of the feto-maternal barrier results in 3: increased maternal plasma concentrations of HbF. Excess oxyHb undergoes auto-oxidation reactions resulting in free heme-groups and formation of ROS. 4: A complex network of scavenger proteins, composed of Hp, Hpx and A1M, binds, inhibits and eliminate HbF, heme and ROS. Cell-free HbF is bound by Hp and cleared by CD163 receptor-mediated uptake in monocytes and macrophage-cells. Free heme-groups are bound by Hpx and heme is cleared via the Hpx receptor CD91, preferably expressed on macrophages and hepatocytes. In this study, a highly significant decrease of both the Hp and Hpx was observed in maternal plasma of women with PE as compare to normal pregnancies. This indicated a prolonged presence of increased levels of both extracellular Hb and heme. Analysis of the plasma A1M levels in the present study displayed a significantly increase in women with PE as compared to normal pregnancies, most likely as a result of oxidative stress-induced up-regulation of the A1M gene expression.

In this study cell-free HbF and the endogenous Hb- and heme-scavenger systems were characterized in pregnant women diagnosed with PE and normal pregnancies. Congruent with previous results, a significant increase of HbF was observed in women with PE in term pregnancies [13]. Furthermore, plasma levels of the Hb- and heme scavenger systems Hp and Hpx were statistically significantly reduced, suggesting an increased consumption. In line with previously published studies [13,14] the extravascular heme- and radical scavenger A1M was significantly increased in plasma of women with PE. The diagnostic and clinical utility of the investigated biomarkers was also evaluated and a clear potential in using these as clinical tools for diagnosing women with PE and predicting the obstetrical outcomes was found. The findings of this paper and a possible chain of events involved in the development of PE are discussed in details below and summarized in Fig 5.

Hemolysis and the subsequent release of cell-free Hb and heme occur in a wide range of clinical conditions and diseases, such as hemorrhage, transfusion reactions, malaria, and sickle cell disease. The release of cell-free Hb and heme causes a range of pathophysiological effects where hemodynamic instability and tissue injury constitutes the major insults [43,44]. Immediate effects include scavenging of the potent vasodilator nitric oxide (NO) that leads to increased arterial blood pressure [45,46]. Furthermore, cell-free Hb and free heme have been described to be accumulated and compartmentalized within the vascular wall causing subsequent organ failure of the kidneys [47,48]. Long-term exposure to cell-free Hb and heme has been described to be associated with NO depletion, inflammation and oxidative stress [44,45]. Thus, inadequate scavenging and protection against extracellular Hb and its metabolites during pregnancy may cause fundamental damage to the vascular bed. In fact, the long-term effects of PE are increased risk of cardiovascular disease and stroke later in life [49,50]. In a series of recent publications the importance of cell-free HbF and its downstream metabolites free heme and ROS, in the development of PE-related damage and symptoms, have been described [12,47,51,52]. By using the dual placenta perfusion system, May et al [12] described placental damaging effects following exposure to cell-free Hb. Hb caused a significant increase in perfusion pressure and damage to the blood-placenta barrier followed by leakage of extracellular Hb into the maternal circulation. Electron microscopy displayed morphological changes similar to what is seen in placentas of women with PE [53]. In the pregnant ewe PE-model, a starvation-induced hemolysis model, increased amount of extracellular heme, bilirubin and ROS in the blood as well as damage to the placenta and kidneys has been shown [51,54,55].

In order to protect ourselves against extracellular Hb and free heme, humans have evolved several Hb- and heme-detoxification systems. Previous studies have shown that the plasma levels of Hp are decreased in PE pregnancies [13,56]. If Hp becomes depleted, as a consequence of consumption due to high levels of cell-free Hb or prolonged exposure time to cell-free Hb, the uncleared oxyHb will undergo auto-oxidation reactions resulting in the formation of metHb, free heme and ROS. Furthermore, the non-Hp bound Hb will be accumulated within organs, predominantly the kidneys, where it causes damage. In renal tissues, the glomeruli are affected, subsequently leading to leakage of proteins into the urine [57]. Upon release of heme into the blood stream, Hpx, a highly specific and abundant heme-scavenger protein that protects blood and endothelial cells against heme-induced damage, binds the heme and forms an Hpx-heme complex that is cleared by macrophages, hepatocytes, neurons and syncytiotrophoblasts expressing the CD91 receptor. Heme is subsequently internalized by endocytosis of the receptor /Hpx-heme unit and heme is degraded by HO-1 [23,24]. HO-1 is a cytosolic enzyme that participate in heme-detoxification by binding and degrading the free heme-group [26] and the expression of HO-1 is induced by a variety of conditions of environmental stress [58,59]. It operates in concert with microsomal NADPH-cytochrome P450 reductase to convert heme to biliverdin, CO and Fe^2+^, utilizing three molecules each of O_2_ and NADPH for each molecule of heme [60–62]. The products of HO-activity have important beneficial physiological activities providing further antioxidation effects besides the mere elimination of heme. Biliverdin is reduced to bilirubin by biliverdin reductase [63] and bilirubin is a powerful physiological antioxidant [64]. CO has been reported to have both pro-oxidant and antioxidant effects, mostly as a result of binding to heme-proteins, replacing molecular O_2_ (reviewed in [65]). Recently, the importance of HO-1 and CO in sustaining pregnancy was reported. Indeed, CO was suggested as a possible therapy for the treatment of PE [66]. This could be explained by the finding that CO induces vasodilation by binding to the heme-protein guanylyl cyclase [67]. In this paper, we have focused on the extracellular Hb-protection proteins Hp, Hpx and A1M.

Studies of sickle cell anemia patients have reported decreased levels of Hpx following hemolysis [68]. Here we observed a statistically significant decrease of both the Hp and Hpx in maternal plasma of women with PE as compared to normal pregnancies, suggesting a prolonged presence of increased levels of both cell-free Hb and heme. This is in line with the previous study by Anderson et al [14], reporting increased serum levels of cell-free Hb as early as the first trimester in women that later developed PE. Interestingly, the Hp levels in women with late onset PE were considerably lower than in women with early onset PE. In addition, some PE patients displayed a significant increase in cell-free HbF (non Hp-bound), and these high levels were only found in women with the Hp 2–2 isoform (Fig 2C). Thus, this sub-group of PE patients may have a reduced innate defense system against cell-free Hb and may constitute a high-risk group. In fact, a majority developed a severe condition of PE (3 out of 7) or had early onset PE (4 out of 7).

Due to scavenging of HbF by Hp it is expected that the maternal plasma samples contain two major forms of HbF: cell-free, non Hp-bound HbF and Hp-HbF. In order to target these, two different ELISA assays for separate quantification of the two molecular species were developed. The results showed significantly increased concentrations of both free HbF and Hp-HbF complex in women with PE but the magnitude of the increase was much less for Hp-HbF than free HbF. As described above, once cell-free HbF appears in blood it is rapidly scavenged by Hp and the Hp-HbF complex is quickly cleared from the blood by internalization in CD163-bearing cells. Therefore, a possible explanation for the small differences in Hp-HbF complex concentrations between the PE and control groups may be that the high turnover-rate of the Hp-HbF complex tends to counteract accumulation of the complex in PE and thus obscures the differences.

Hpx concentration was shown to have a significant negative correlation to the blood pressure (Fig 3). It could be speculated that this is also correlated to the severity of the disease, although no statistical significance was seen between Hpx and blood pressure when separating PE patients from controls. Previous studies have shown that enzymatically active Hpx can affect the renin-angiotensin system (RAS) in *in vitro* by downregulating the vascular angiotensin II receptor (AT(1)) and promoting an expanded vascular bed [69,70]. It could be speculated that increased heme levels, resulting from elevated levels of cell-free HbF, in women with PE leads to a consumption of Hpx and consequently a reduced Hpx activity, resulting in an enhanced AT(1) receptor expression and a contracted vascular bed. In fact, Bakker et al [71] showed that plasma from women with PE had an increased AT(1) receptor expression on monocytes as compared with plasma from normal pregnancies. This, together with NO consumption, may be important blood pressure regulating effects caused by elevated extracellular HbF observed in PE.

We have previously shown that the radical scavenger A1M binds and degrades heme [28,29,72]. Addition of the protein protects cells and tissues against oxidative insult, structural- and functional damage and prevents cell death [12,32,73,74]. In line with previous studies [13,14] the A1M plasma concentration was shown to be significantly increased in women with PE. This increase was statistically significant in women with late onset PE, but not in women with early onset PE.

Why are the A1M-levels increased while the Hp- and Hpx-levels are decreased in the PE patients? Several reports describe that the A1M gene expression is rapidly upregulated in the liver, skin, placenta and other organs as a response to increased levels of Hb, heme and ROS [12,13,74]. This will lead to increased secretion of the protein resulting in increased plasma concentrations in pathological situations with increased Hb and ROS loads [57]. Furthermore, no specific receptor-mediated clearance system of A1M has been shown to be triggered during hemolysis or oxidative stress, whereas Hp and Hpx are cleared from plasma upon binding to Hb and heme [24,25]. As a result, the concentrations of A1M in plasma and extravascular fluids will increase, while Hp and Hpx will be exhausted and hence their plasma concentrations will decrease.

There is an increased attention towards the use of biomarkers in clinical prediction and diagnosis of PE [35,75,76]. Several biomarkers have been suggested but so far, but no available guidelines recommend the use of biomarkers in clinical screening programs [6–8]. Recently, the American College of Obstetricians and Gynecologists (ACOG) suggested that the definition of severe PE should replace proteinuria by the use of biomarkers; thrombocytes (<100,000/microliter), serum creatinine (>1.1 mg/dl) and liver transaminases (twice the normal concentration) [76]. In this study, we present data suggesting that HbF, Hpx and A1M can be used as clinical biomarkers in supporting the diagnosis of PE. The combination of HbF, Hpx and A1M displayed the highest correlation to diagnosis (detection rate of 69% at 5% false positives, AUC = 0.88, Fig 4A) and the combination of Hpx and A1M also displayed a high detection rate (66% at 5% false positive, AUC = 0.87, Fig 4B). Thus, HbF, Hpx and A1M constitute possible future markers that could support the diagnosis of PE.

Being able to predict fetal and maternal outcomes is of great clinical value as it can help clinicians in the difficult task to optimize timing of delivery and mobilize neonatal resources. In this study the correlation between investigated biomarkers and a range of maternal and fetal outcomes were evaluated. The results indicated that HbF, Hp and Hpx correlated with admission to NICU. Furthermore, Hpx was strongly associated to premature birth. However, since all prematurity in this cohort was associated with PE this strong association could be as result of the strong correlation between Hpx and PE rather than prematurity itself.

It is of importance to note that the cohort used in this case-control study contains an over-representation of women with PE. Consequently, detection and prediction rates reported in this study will most likely be different than in a normally distributed cohort, containing 3–8% of PE cases. Studies on such normally distributed, and larger, cohorts have been initiated.

In summary, we have characterized cell-free HbF and the endogenous Hb- and heme-scavenger systems in pregnancies complicated by PE. Plasma levels of HbF were significantly elevated whereas Hp and Hpx were significantly decreased in women with PE. The extravascular heme- and radical scavenger, and marker of oxidative stress, A1M was significantly increased in plasma of women with PE. Furthermore, HbF and the related scavenger proteins displayed a potential to be used as clinical biomarkers for more precise diagnosis of PE and as predictors that help identifying pregnancies with increased risk of obstetrical complications.

## List of Abbreviations

A1M: α_1_-microglobulin
ACOG: American College of Obstetricians and Gynecologists
AT(1): Angiotensin II Receptor
AUC: Area Under the ROC-curve
CD163: Cluster of Differentiation 163
Fe^2+^: Ferrous Iron
Fe^3+^: Ferric Iron
Fe^4+^: Ferryl
GPx: Glutathione Peroxidase
Hb: Hemoglobin
HbA: Adult Hemoglobin
HbF: Fetal Hemoglobin
HELLP: Hemolysis, Elevated Liver Enzymes and Low Platelets Count
HO: Heme Oxygenase
Hp: Haptoglobin
Hp-Hb: Haptoglobin-Hemoglobin Complex
Hp-HbF: Haptoglobin-Fetal Hemoglobin Complex
Hpx: Hemopexin
HRP: Horseradish Peroxidase
IUGR: Intrauterine Growth Restriction
LF: Low Fluorescence
MetHb: Ferric (Fe^3+^) Hemoglobin
NICU: Neonatal Intensive Care Unit
NO: Nitric Oxide
O_2_: Oxygen
PE: Preeclampsia
PVDF: Polyvinylidene Difluoride
RAS: Renin-Angiotensin System
ROC: Receiver Operational Curve
ROS: Reactive Oxygen Species
RT: Room Temperature
SOD: Superoxide Dismutase
SPSS: Statistical Package for the Social Sciences
TMB: 3,3',5,5'-Tetramethylbenzidine
