# Correction: The Human Endogenous Protection System against Cell-Free Hemoglobin and Heme Is Overwhelmed in Preeclampsia and Provides Potential Biomarkers and Clinical Indicators

**DOI:** 10.1371/journal.pone.0229816

**Published:** 2020-02-26

**Authors:** Magnus Gram, Ulrik Dolberg Anderson, Maria E Johansson, Anneli Edström-Hägerwall, Irene Larsson, Maya Jälmby, Stefan R. Hansson, Bo Åkerström

In the funding statement, the Swedish Foundation for Strategic Research (RBP14-0055) is missing as a funding source.

The correct funding statement is as follows: This work was supported by The Swedish Research Council, Swedish Foundation for Strategic Research (RBP14-0055), Region Skåne (ALF), the Marianne & Marcus Wallenberg Foundation, the Torsten Söderbergs Foundation, the Maggie Stephens Foundation, the Greta and Johan Kocks Foundation, the Fanny Ekdahls Foundation, the Crafoordska Foundation, the Österlunds Foundation and A1M Pharma. The funders had no role in study design, data collection and analysis, decision to publish, or preparation of the manuscript.
